# Oxidative Pentose Phosphate Pathway Enzyme 6-Phosphogluconate Dehydrogenase Plays a Key Role in Breast Cancer Metabolism

**DOI:** 10.3390/biology10020085

**Published:** 2021-01-23

**Authors:** Ibrahim H. Polat, Míriam Tarrado-Castellarnau, Rohit Bharat, Jordi Perarnau, Adrian Benito, Roldán Cortés, Philippe Sabatier, Marta Cascante

**Affiliations:** 1 Department of Biochemistry and Molecular Biomedicine and Institute of Biomedicine (IBUB), Faculty of Biology, Universitat de Barcelona, Av Diagonal 643, 08028 Barcelona, Spain; polat@med.uni-frankfurt.de (I.H.P.); mtarrado@ub.edu (M.T.-C.); rohitbharat1990@gmail.com (R.B.); jperarnau33@gmail.com (J.P.); a.benito-mauricio@imperial.ac.uk (A.B.); roldancg@gmail.com (R.C.); 2 Equipe Environnement et Prédiction de la Santé des Populations, Laboratoire TIMC (UMR 5525), CHU de Grenoble, Université Grenoble Alpes, 38700 CEDEX La Tronche, France; philippe.sabatier@univ-grenoble-alpes.fr; 3 Department of Medicine, Hematology/Oncology, Goethe-University Frankfurt, 60590 Frankfurt, Germany; 4 Centro de Investigación Biomédica en Red de Enfermedades Hepáticas y Digestivas (CIBEREHD), Instituto de Salud Carlos III (ISCIII), 28001 Madrid, Spain; 5 Division of Cancer, Department of Surgery and Cancer, Faculty of Medicine, Imperial College London, London W12 0NN, UK

**Keywords:** breast cancer, pentose phosphate pathway, cancer metabolism, 6PGD

## Abstract

**Simple Summary:**

Cancer cells alter their metabolism to maintain their high need for energy, produce enough macromolecules for biosynthesis, and preserve their redox status. The investigation of cancer cell-specific metabolic alterations has vital importance to identify targets to be exploited for therapeutic development. The pentose phosphate pathway (PPP) is often highly activated in tumor cells to maintain redox level, as this pathway takes an important role in reactive oxygen species detoxification. PPP also yields ribose-5-phosphate, a five-carbon sugar essential for synthesizing nucleotides necessary for DNA replication and cell proliferation. In this study, we inhibited one of the key enzymes of this biochemical pathway and observed the main functions of this enzyme in breast cancer cells. We have demonstrated that inhibition of this enzyme reduces cell proliferation and leads to cell cycle arrest and apoptosis. Besides that, we showed that the inhibition of this enzyme causes an alteration in cellular metabolism. All these findings indicate that targeting this enzyme with specific pharmacological inhibitors is an effective strategy in fighting cancer.

**Abstract:**

The pentose phosphate pathway (PPP) plays an essential role in the metabolism of breast cancer cells for the management of oxidative stress and the synthesis of nucleotides. 6-phosphogluconate dehydrogenase (6PGD) is one of the key enzymes of the oxidative branch of PPP and is involved in nucleotide biosynthesis and redox maintenance status. Here, we aimed to analyze the functional importance of 6PGD in a breast cancer cell model. Inhibition of 6PGD in MCF7 reduced cell proliferation and showed a significant decrease in glucose consumption and an increase in glutamine consumption, resulting in an important alteration in the metabolism of these cells. No difference in reactive oxygen species (ROS) production levels was observed after 6PGD inhibition, indicating that 6PGD, in contrast to glucose 6-phosphate dehydrogenase, is not involved in redox balance. We found that 6PGD inhibition also altered the stem cell characteristics and mammosphere formation capabilities of MCF7 cells, opening new avenues to prevent cancer recurrance after surgery or chemotherapy. Moreover, inhibition of 6PGD via chemical inhibitor S3 resulted in an induction of senescence, which, together with the cell cycle arrest and apoptosis induction, might be orchestrated by p53 activation. Therefore, we postulate 6PGD as a novel therapeutic target to treat breast cancer.

## 1. Introduction

Alteration in metabolism is one of the emerging hallmarks of cancer [1]. Many observations made during the early period of cancer biology research revealed metabolic changes, such as the Warburg effect [2,3], to be a common feature of cancer cells. Nowadays, multiple molecular mechanisms, both intrinsic and extrinsic, are known to play a vital role in reprogramming cellular metabolism in order to fulfill the three basic needs for cancer cell survival: (i) maintenance of energy status, (ii) increased biosynthesis of macromolecules, and (iii) preservation of appropriate redox status. Nevertheless, many mechanisms regulating metabolic reprogramming are still unknown, and the search for novel tumor-specific metabolic dependencies exploitable for therapeutic interventions is a growing field in cancer research.

The pentose phosphate pathway (PPP) plays essential roles not only in nucleotide biosynthesis and the maintenance of redox status but also in various aspects related to cancer cells viability, including proliferation, apoptosis, drug resistance, invasiveness, metastasis, and senescence [4,5,6,7,8]. Moreover, as an alternative pathway to glycolysis for glucose metabolism, PPP metabolizes from 5 to 30% of glucose depending on the tissue type [8]. In particular, breast cancer cells are reported to be eight times more dependent on PPP compared to their non-cancerous counterparts to maintain their redox status [9]. Because of all these crucial aspects, cancer cells are significantly dependent on PPP to maintain their highly proliferative state [10,11]. Therefore, the therapeutic potential of targeting PPP has attracted the interest of researchers in the field and both oxidative and non-oxidative branches of this metabolic pathway have been of keen interest to be used as a therapeutic drug target [5,10,12,13].

PPP consists of two branches that converge in the production of ribose-5-phosphate, which is essential for the synthesis of nucleotides (Supplementary Figure S1). The oxidative branch of PPP (ox-PPP) is a non-reversible metabolic pathway where glucose-6-phosphate (G6P) is transformed into 6-phosphoglucono-δ-lactone by glucose-6-phosphate dehydrogenase (G6PD) and, subsequently, to ribulose-5-phosphate by 6-phosphogluconate dehydrogenase (6PGD) with the concomitant production of nicotinamide adenine dinucleotide phosphate (NADPH). The resulting ribulose-5-phosphate is then converted to ribose-5-phosphate and used for biosynthesis of nucleotides. The non-oxidative branch (nonox-PPP), on the other hand, consists of a set of reversible reactions in which ribose-5-phosphate and xylulose-5-phosphate are converted to glyceraldehyde-3-phosphate and fructose-6-phosphate by transketolase (TKT) and transaldolase (TALDO), which are then utilized for glycolysis [8,14].

The oxidative branch of PPP is of particular interest because it is able to produce NADPH, and therefore plays a key role in the regulation of reactive oxygen species (ROS) levels. The balance between ROS production and scavenging has been demonstrated to be altered in some tumors and has also been proposed to be an attractive therapeutic target on its own [9,15]. 6PGD, the third enzyme of the ox-PPP, is an excellent target since it plays an essential role in both the synthesis of nucleotides and generation of NADPH required for ROS detoxification. Although the importance of 6PGD had till now been overshadowed by G6PD and TKT [16,17], recent evidence suggests that G6PD alone has only marginal effects on in vitro proliferation of some particular types of cancer cells, including breast cancer [6,18,19]. Moreover, new evidence highlighting the potential of 6PGD as a promising therapeutic target in certain forms of cancer has emerged [6,20,21,22].

We suggest 6PGD as a better target than G6PD for various reasons. First, despite the fact that the entire PPP is regulated by several factors, G6PD is much more tightly controlled through several levels involving transcriptional, translational, post-translational, and metabolic regulations, being the rate-limiting enzyme of this pathway [23]. Besides, the NADPH/NADP+ ratio is an important regulator of G6PD, and since up to 75% NADPH required for de novo lipid synthesis in cellular organisms is produced by G6PD, this enzyme is also considered as a part of the lipogenic enzyme family, and it is further regulated by hormone and nutritional factors [20,24]. Therefore, altering the function of G6PD is more challenging than other PPP enzymes. On the other hand, inhibition of 6PGD leads to the accumulation of 6-phosphogluconate (6-PG), a critical glycolysis regulator [24]. Glycolytic enzyme phosphoglycerate mutase 1 (PGAM1) is reciprocally regulated by 6-PG in a way that 3-phosphoglycerade (3-PG), the substrate of this enzyme, is also a competitive inhibitor of 6PGD [24]; therefore, accumulation of 6-PG affects the activity of PGAM1. Besides that, recent studies reported the direct relationship of 6PGD and the AMP-activated protein kinase (AMPK) signaling pathway [20,25,26,27,28], which further shows the importance of targeting this enzyme in cancer cells.

In our study, we first characterized the metabolic reprogramming induced by the knockdown of 6PGD by RNAi-mediated silencing to further explore the potential of this enzyme as a therapeutic drug target in the MCF7 breast cancer cell line. Then, we used a chemical selective 6PGD inhibitor, S3 (1-hydroxy-8-methoxy-anthraquinone), to test the effect of the inhibition of 6PGD on cellular senescence. In addition, we aimed to investigate the relation between p53 activation and PPP, since p53 has several vital functions in cellular metabolism, among which are cell cycle regulation, apoptosis induction, regulation of glycolysis and PPP, and cellular senescence [29,30,31]. Because of the high reliance of breast cancer cells on PPP and their dependence on ROS detoxification to manage oxidative stress and maintain their survival [11,22,32], we targeted 6PGD in MCF7. MCF7 is an estrogen and progesterone receptor (ER and PR, respectively) positive, and a human epidermal growth factor receptor 2 (HER2) negative cell line [33]. This cell line is demonstrated to have more than 4 times higher 6PGD at mRNA, protein, and enzyme activities levels compared to healthy breast cancer cell lines MCF10A and HMT3522. Besides that, it shows similar 6PGD levels at mRNA, protein, and specific enzyme activity levels among a panel of different breast cancer cell lines including MDA-MB-468 and MDA-MB-231 (both triple negative) and SKBR3 (ER, PR negative, and HER2 positive) [22,33].

## 2. Materials and Methods

### 2.1. Cell Culture

Breast cancer cell line MCF7 was purchased from ATCC, and it was cultured in MEM without phenol red (Gibco, Thermo Fisher Scientific, Waltham, MA, USA) containing 10% fetal bovine serum (Gibco, Thermo Fisher Scientific, Waltham, MA, USA), 10 mM d-glucose (Sigma-Aldrich, St. Louis, MO, USA), 1 mM sodium pyruvate (Biological Industries, Beit HaEmek, Israel), 2 mM glutamine (Gibco, Thermo Fisher Scientific, Waltham, MA, USA), 0.1% antibiotic (penicillin 10 units/mL-streptomycin 10 units/mL, Gibco), 0.01 mg/mL insulin (Sigma, St. Louis, MO, USA), and 1% non-essential amino acids (Biological Industries, Beit HaEmek, Israel). Cells were maintained at 37 °C with 5% CO^2^ and saturated humidity. Growth medium was replaced every 2–3 days, and cells were passed before they reached 80% confluence.

### 2.2. siRNA Transfection

For the transfection, MCF7 cells were seeded at a density of 1 × 10^5^ cells per well in a 6-well plate with an antibiotic-free growth medium. After 24 h, cells were transfected in triplicates with 50 nM of either siNEG or siRNAs against 6PGD using Metafectene^®^ Pro (Biontex, München, Germany) according to the manufacturer’s protocol. The medium was replaced after 6 h with normal medium containing antibiotics. The siRNA sequences targeting 6PGD were purchased from Dharmacon (Lafayette, CO, USA) and are listed as follows: si6PGD-1, GAUCAUCUCUUACGCUCAA; si6PGD-2, GAGCAGGCCACUUCGUGAA. Non-targeting siRNA (siNEG) was also purchased from Dharmacon (sequence not provided by the manufacturer).

### 2.3. RNA Isolation and Gene Expression Analysis

RNA isolation from the transfected cells from fresh or frozen plates was done using Trizol^®^ reagent (Sigma, MA, USA) according to the manufacturer’s protocol. Conversion of RNA into cDNA was done using 1 μg of RNA, random primers (Roche, Basel, Switzerland), and M-MLV reverse transcriptase enzyme (Invitrogen) according to the manufacturer’s protocol. Gene expression analysis was performed by RT-PCR (Applied Biosystems^®^ 7500 Real Time PCR) in standard conditions provided by the manufacturer employing Taqman^®^ (Applied Biosystems, Thermo Fisher Scientific, Darmstadt, Germany) gene-specific probes for 6PGD. The expression levels were quantified using the ΔΔCt method using peptidylprolyl isomerase A (PPIA) as a reference gene.

### 2.4. Enzyme Activity Assays

Fresh cell culture plates were rinsed with PBS and lysed with lysis buffer (20 mM tris-HCl, pH 7.5, 1 mM dithiothreitol, 1 mM EDTA, 0.02% (*v*/*v*) triton X-100, 0.02% (*v*/*v*) sodium deoxycholate) supplemented with protease and phosphatase inhibitor cocktails (Thermo Scientific). Cells were scrapped and the cell lysate was disrupted by sonication using a titanium probe (Vibracell, Sonics & Materials Inc., Tune 50, Output 20, 3 cycles of 5 s each) and centrifuged at 12,000× *g* at 4 °C for 20 min. The supernatant was separated and immediately used to determine specific enzyme activities of 6-phosphogluconate dehydrogenase, lactate dehydrogenase, malic enzyme, isocitrate dehydrogenase, and transketolase using the COBAS Mira Plus analyzer (Horiba ABX, Kyoto, Japan). All enzymatic activities were determined by monitoring the increase or decrease of absorbance due to NAD(P)H at 340 nm wavelength (ε 6.23 × 10^3^). The enzyme activity for each sample was then normalized to the total protein content of the samples measured by BCA assay at 550 nm (Pierce, Thermo Fisher Scientific, Waltham, MA, USA).

#### 2.4.1. 6-Phosphogluconate Dehydrogenase (6PGD), Malic Enzyme (ME) and Isocitrate Dehydrogenase (IDH)

Specific activities of 6PGD, ME, and IDH were measured by adding samples to a cuvette containing 0.5 mM NADP+ in 50 mM tris-HCl (including 0.2 mM MgCl_2_), pH 7.6, at 37 °C. The reaction was initiated by the addition of 6PG, malate or isocitrate, respectively, up to a final concentration of 2 mM.

#### 2.4.2. Lactate Dehydrogenase

LDH specific activity was measured by adding samples to a cuvette containing 0.2 mM NADH in 100 mM KH_2_PO_4_/K_2_HPO_4_, pH 7.4, at 37 °C. The reaction was initiated by the addition of 10 mM pyruvate up to a final concentration of 0.2 mM.

#### 2.4.3. Transketolase

Specific activity of TKT was measured by adding samples to a cuvette containing 5 mM MgCl_2_, 0.2 U/mL triose phosphate isomerase, 0.2 mM NADH, 0.1 mM thiamine pyrophosphate in 50 mM tris-HCl, pH 7.6, at 37 °C. The reaction was initiated by the addition of a substrate mixture prepared by dissolving 50 mM ribose-5-phosphate in 50 mM tris-HCl, pH 7.6, in the presence of 0.1 U/mL ribulose-5-phosphate-3-epimerase and 1.7 mU/mL phosphoriboisomerase.

### 2.5. Cell Proliferation and Cell Cycle Distribution Analysis

Proliferation kinetics and viability of the transfected cells were measured using flow cytometry combining direct cell counting and propidium iodide (PI) staining. The analysis was performed using a Beckman Coulter^®^ Epics^®^ XLTM Flow Cytometer adjusted to 1 × 10^4^ fluorospheres cut-off. Total cell number was registered, allowing discrimination between dead and alive cells.

For cell cycle analysis, the transfected cells were harvested after 96 h, resuspended in 200 μL of 1X TBS buffer, fixed and stained with 200 μL of vindelov-PI solution, and incubated at room temperature for 30 min in the dark. The analysis was performed using a Beckman Coulter^®^ Epics^®^ XLTM Flow Cytometer with a cut-off at 1 × 10^4^ cells. Cell cycle distribution analysis was done using FlowJo^®^ software, through which the percentage of cells in G1, S, and G2 phases was obtained.

Viability assay was performed by a modification of the 3-(4,5-dimethylthiazol-2-yl)-2,5-diphenyltetrazolium bromide (MTT) assay described by Mosmann et al. [34]. Briefly, 3 × 10^3^ MCF7 cells/well were seeded in 96-well plates for 24 h before exposure with different concentrations of S3 (Sigma-Aldrich, MA, USA), and serial dilutions of the compound was added to cell medium. DMSO was added to the control to ensure the real dose response of the tested compound. Cells were incubated at 37 °C with 5% CO_2_ for 72 h. Then, the medium was removed, and 100 µL of filtered MTT in PBS (0.5 mg mL^−1^) was added to wells. After 1 h of incubation with MTT, the medium containing MTT was aspirated, and the formazan product was dissolved in 100 µL of DMSO. Absorbance values were measured in an ELISA plate reader at 550 nm wavelength and normalized to the absorbance of cells cultured without compound. The inhibitory concentration values were determined using GraphPad Prism software.

### 2.6. Mammosphere Formation Assay (3D Cell Culture)

The capability of MCF7 cells to grow as single-cell colonies in low-attachment conditions was analyzed by mammosphere formation assay. siRNA transfection was performed as described before, and the cells were trypsinized 24 h after transfection. Then, cells were reseeded at a density of 7500 cells/well into 24-well ultra-low attachment plates (Corning Costar, Corning, NY, USA) along with 1.5 mL of complete media supplemented with 20 ng mL^−1^ EGF, 20 ng mL^−1^ bFGF, 10 µg mL^−1^ heparin, B27 (1:50), and 0.5 µg mL^−1^ hydrocortisone. The cells were allowed to grow undisturbed for 10 days and then checked under a microscope for the mammosphere formation. The quantification of mammosphere formation capability was measured using the MTT assay [34] by adding MTT reagent to a final concentration of 0.5 mg/mL into each well followed by incubation at 37 °C for 2 h. The plates were then scanned using the HP ScanJet G-4010 scanner at a pixel density of 2400 ppp and analyzed using ImageJ^®^ Software (public domain National Institutes of Health, USA, http://rsbweb.nih.gov/ij/). Quantification of the mammosphere formation capability was done by calculating the total surface area occupied by mammospheres with a size greater than 0.0000785 cm^2^, corresponding to a spheroid diameter of 0.01 mm.

### 2.7. Western Blot

Protein extracts were obtained from either fresh or frozen plates 96 h after transfection using the protocol described for the enzyme activity assays. The protein level in each sample was quantified using the BCA assay according to the manufacturer’s protocol. Western blot analysis was carried out using 30 μg of protein, and after electrophoretic separation, proteins were transferred onto a PVDF membrane. The membranes were then blocked with 0.5% of non-fat dry milk in 0.1% PBS-Tween, and then incubated with p53 polyclonal (Merck, Millipore, Darmstadt, Germany) antibody followed by exposure to corresponding horseradish peroxidase-conjugated secondary antibody. Visualization was carried out on Fujifilm X-ray using chemiluminescence detection.

### 2.8. Metabolite Production and Consumption Analysis

Glucose consumption rates in the cells were analyzed by measuring the decrease in the concentration of extracellular glucose in the media at 96 h, compared to the initial concentration of glucose, with respect to the total cell number at each time point. The extracellular glucose concentration at a given time point was measured by calculating the decrease in NAD(P)H concentration caused by the conversion of total glucose by hexokinase and conversion of resulting glucose-6-phosphate into D-gluconate-6-phosphate by G6PD. Similarly, the glutamine consumption rate was estimated by measuring the change in NADH concentration after conversion of extracellular glutamine into glutamate by glutaminase and the following conversion of glutamate to α-ketoglutarate by glutamate dehydrogenase. Glutaminase reaction was performed by incubating the media samples with 125 mU mL^−1^ glutaminase in 125 mM acetate buffer (pH 5) with soft agitation for 30 min at 37 °C. Lactate concentration was determined by measuring the change in NADH concentration resulting from the lactate dehydrogenase (LDH) reaction, which was carried out at 37 °C by adding media to a cuvette containing 1.22 mg/mL NAD and 87.7 U/mL LDH in 0.2M hydrazine 12 mM EDTA buffer, pH 9. All the measurements were done using a COBAS Mira Plus chemistry analyzer (ABX Diagnostics) and measuring the absorbance of NAD(P)H at 340 nm.

### 2.9. Intracellular ROS Level Measurement

Total intracellular ROS levels were determined using flow cytometry and an H2DCFA probe (Sigma, MA, USA). Cells were incubated with 5 μM H2DCFA in PBS for 30 min. Afterwards, PBS was replaced with complete growth medium, and cells were incubated for 15 min at 37 °C and 5% CO_2_. Next, cells were trypsinized and resuspended in a solution containing 50 μM H2DCFA and 20 μg/mL propidium iodide. Internalized probes reacted with ROS and emitted fluorescence when excited at 492 nm. Emitted fluorescence was recorded by a flow cytometer (Beckman Coulter^®^ Epics^®^ XLTM) at 520 nm wavelength with a cutoff range of 1 × 10^4^ cells. For the ROS analysis, only PI-negative cells were taken into consideration.

### 2.10. Apoptosis Measurement

The apoptosis measurement was conducted as previously described [35]. In short, the transfected cells were washed twice with PBS and trypsinized. The cells were then collected and incubated in 195 μL of 1X binding buffer (10 mM hepes/NaOH, pH 7.4, 140 mM NaCl, 2.5 mM CaCl_2_) and 5 μL of annexin V (BendersMedSystem, Vienna, Austria) for 30 min in the dark. Then, 10 μL of 20 μg/mL PI was added to the samples before analysis, and the percentage of apoptotic cells was measured using the Beckman Coulter^®^ Epics^®^ XLTM Flow Cytometer with a cutoff range of 1 × 10^4^ cells. Data analysis was carried using FlowJo^®^ Software.

### 2.11. Senescence-Associated-β Galactosidase Activity

We used the senescence β-galactosidase staining kit (9860S; Cell Signaling Technologies, Danvers, Massachusetts, USA) to detect β-galactosidase activity at pH 6.0 following the manufacturer’s instructions. In brief, cells were fixed for 15 min at room temperature and then incubated with β-galactosidase staining solution containing X-gal in a dry incubator (without CO_2_) at 37 °C overnight. As the blue color developed, images were taken using a contrast phase microscope.

### 2.12. Statistical Analysis

For statistical analysis, parametric unpaired two-tailed independent samples Student’s t-test (Mann–Whitney U test) was used. In all figures, bars represent the mean of triplicates ± SD. Statistical significance was assumed if a null hypothesis could be rejected when at least *p* < 0.05 for a confidence interval of >95%. One asterisk (*) denotes *p*-value < 0.05, two asterisks (**) denote *p*-value < 0.01 and three asterisks (***) denote *p*-value < 0.001.

## 3. Results

### 3.1. 6PGD Was Successfully Inhibited in MCF7 Cells

**Figure 1:**
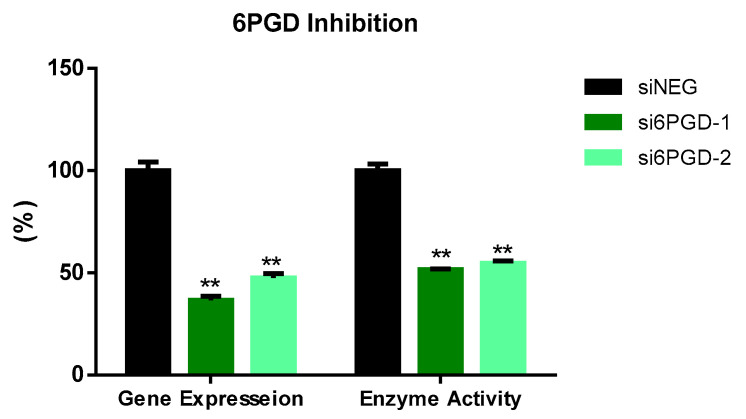
6PGD mRNA expression 72 h after transfection with non-targeting siRNA (siNEG) or siRNAs against 6PGD. Fold change was calculated with respect to siNEG. 6PGD enzyme activity levels 96 h post-transfection using either siRNAs against 6PGD or non-targeting siRNA. Two asterisks (**) denote *p*-value < 0.01.

We tested the functional role of 6PGD in breast cancer using MCF7 cells as a cell model.Two siRNA sequences targeting different exonic regions of the 6PGD gene were tested and the knockdown of 6PGD was measured at mRNA level 72 h after transfection. Both siRNA sequences showed successful knockdown of 6PGD mRNA expression on MCF7 cells, with a decrease in fold change of around 60% when compared to the cells transfected with siNEG. Similarly, the reduction of 6PGD activity was observed 96 h after transfection by measuring specific enzyme activity. Correspondingly, both siRNA sequences induced a decrease of around 50% of enzyme activity in MCF7 cells (Figure 1).

### 3.2. 6PGD Knockdown Affects Cell Proliferation, Cell Cycle Distribution, and Apoptosis

**Figure 2:**
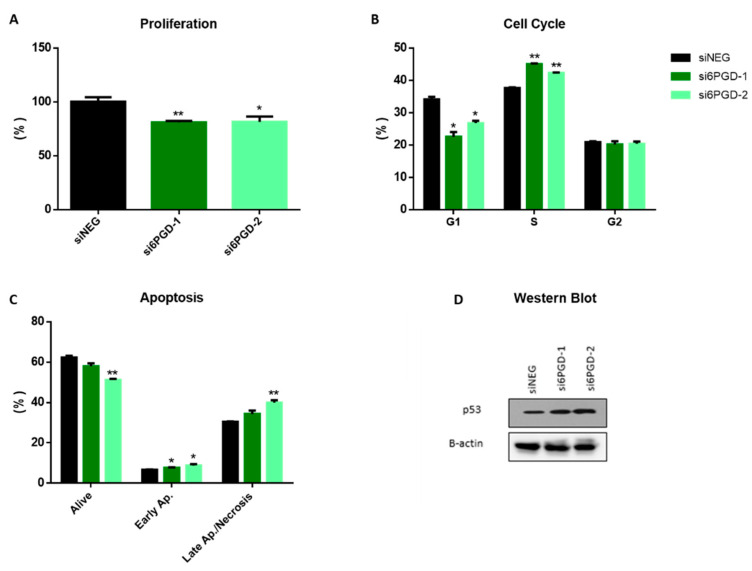
(**A**) Flow cytometry measurements of cell proliferation, 96 h after transfection, for 6PGD-knockdown cells and cells transfected with siNEG. (**B**) Cell cycle distribution analysis of MCF-7 cells after 96 h of siRNA transfection. The percentage of cells in the different cell cycle phases was calculated using FlowJo^®^ software. (**C**) Apoptosis analysis of MCF7 cells after 96 h of siRNA transfection, measured by flow cytometry using annexin V FITC kit, 96 h after transfection. The percentage of cells in the different apoptosis phases was calculated using FlowJo^®^ software. (**D**) Western blot analysis of p53 expression in cells transfected with si6PGD vs. cells transfected with siNEG 96 h after transfection. One asterisk (*) denotes *p*-value < 0.05, two asterisks (**) denote *p*-value < 0.01.

The effect of 6PGD knockdown on the cell proliferation rate was measured via flow cytometry, combining direct cell counting and propidium iodide (PI) staining. Then, 96 h after transfection, 6PGD-knockdown MCF7 cells proliferated around 25% less compared to siNEG-transfected cells (Figure 2A). This result proves that the altered cellular metabolism due to 6PGD inhibition results in reduction in cell proliferation rates. It is worth noting that this effect on MCF7 cells’ proliferation rates results from a limited decrease of 6PGD’s activity (less than 50%), which highlights the importance of 6PGD activity in breast cancer cell proliferation. This could be explained because 6PGD inhibition entails a decrease in pentose biosynthesis, which is essential for proliferating cells to synthesize DNA. Besides, the knockdown of 6PGD will also produce an accumulation of 6-phosphogluconate (6PG), which has been reported to cause a slower proliferation of cells by a not yet fully elucidated mechanism [6]. Therefore, a permanent and stronger inhibition of 6PGD is expected to result in greater reductions of the proliferation kinetics of breast cancer cells.

Since both cell cycle arrest and apoptosis induction can be related to decreased cell proliferation rates, we decided to see whether 6PGD knockdown had any effect on them. As it is reported in many studies, cell cycle dysregulation is a hallmark of cancer cells, and several proteins controlling the phases of cell cycle have been proposed as antitumor targets [36,37]. To study the effect of 6PGD knockdown on cell cycle distribution, cells were stained with Vindelov-PI solution after fixation and analyzed by flow cytometry to quantify their DNA content. The analysis of cell cycle distribution indicated a significant arrest in S phase 96 h after 6PGD inhibition (Figure 2B). As any alteration in cell cycle may induce apoptosis [38], we checked whether the arrest in cell cycle induced apoptosis in our cell line by carrying out apoptosis assays 96 h after inhibiting 6PGD gene expression. Flow cytometry analysis using annexin V and PI can assess the presence of non-apoptotic cells (no interaction with either annexin V or PI), early apoptotic cells (interaction with only annexin-V), and late apoptotic or necrotic cells (interaction with both annexin V and PI) [35,39]. In our study, the knockdown of 6PGD in MCF7 showed a significant increase in the number of early apoptotic and late apoptotic and necrotic cells (Figure 2C).

### 3.3. p53 Is Upregulated with 6PGD Knockdown

The tumor suppressor gene p53 has been proven to have effects on the regulation of cell cycle arrest and apoptosis [40]. Considering this, we decided to assess whether the inhibition of 6PGD activity could affect p53 expression. Our results showed an increase in p53 protein levels after the inhibition of 6PGD gene expression in MCF7 (Figure 2D, Supplementary Figure S2A). MCF7 cells are known to have the wild type p53 gene [41], and therefore we hypothesize that p53 overexpression resulting from 6PGD inhibition can lead to cell cycle arrest and apoptosis induction, slowing down proliferation rates in MCF7 cells. Therefore, these results highlight an interesting connection between 6PGD activity and p53 expression, although further studies will be needed to elucidate the relationship between these proteins in a more detailed way.

### 3.4. NADPH Produced by 6PGD Is Dispensable for ROS Detoxification

**Figure 3:**
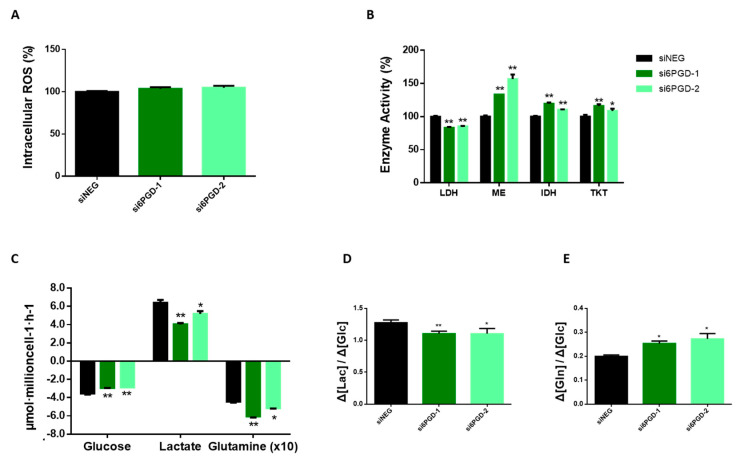
(**A**) Relative total intracellular reactive oxygen species (ROS) level measured by flow cytometry using H2DCFA probes. ROS levels are expressed as fold change with respect to siNEG ROS levels. (**B**) Lactate dehydrogenase, malic enzyme, isocitrate dehydrogenase, and transketolase activity levels measured at 96 h post-transfection using either siRNAs against 6PGD or non-targeting siRNA. Fold changes were calculated as a percentage relative to enzyme activity levels per mg of protein in siNEG-transfected cells. (**C**) Glucose and glutamine consumption and lactate production rates were measured 96 h after transfection by either si6PGD or siNEG. (**D**) Glycolytic activity showing the conversion rate of glucose to lactate. (**E**) Consumption rate of glutamine over glucose. One asterisk (*) denotes *p*-value < 0.05, two asterisks (**) denote *p*-value < 0.01.

The NADPH produced in PPP provides reducing equivalents for protecting the cells against the toxicity of ROS by regenerating reduced glutathione (GSH) from glutathione disulfide (GSSH) by means of glutathione reductase [8]. Taking this into account, we speculated that inhibiting 6PGD, which is involved in NADPH production, might provoke changes in breast cancer cells’ total ROS levels. However, the measurement of total intracellular ROS using H2DCFA probes in MCF7 cells transfected with si6PGD did not show any significant changes in their ROS production compared to cells transfected with siNEG (Figure 3A). This result indicates that 6PGD might not be involved in the maintenance of the redox status of these cells or that these cells are able to resort to alternate pathways to maintain their required NADPH levels. Nevertheless, our results indicate that the antiproliferative activity resulting from 6PGD inhibition is not related to the existence of an enhanced oxidative stress.

### 3.5. 6PGD Inhibition Leads Breast Cancer Cells to Reprogram Their Central Carbon Metabolism

Cancer is not only a disorder of proliferation but also a metabolic disease. Therefore, metabolic changes in cancer cells are considered to have a vital role in explaining tumor formation. Analysis of consumption and production rates of extracellular glucose and lactate showed a significant decrease in glucose consumption 96 h after knockdown of 6PGD gene (Figure 3C). Similarly, lactate production was observed to be reduced, but at a slightly higher rate. Accordingly, Figure 3D shows the glycolytic efficiency decrease of both cell lines with 6PGD knockdown, calculated as moles of lactate produced per moles of glucose consumed, as described previously [42,43]. A decreased rate of glucose to lactate could be due to a shift from glycolysis towards the TCA cycle for the production of energy and other intermediates needed for bioenergetic reactions and cells’ survival. This result is concordant with 6PGD knockdown reducing LDH enzyme activity, as shown in Figure 3B.

Glutamine is an abundant and important amino acid that plays a crucial role in energy generation, redox homeostasis, synthesis of macromolecules, nitrogen metabolism, and several signaling pathways relevant in cancer cells [44,45]. Therefore, we decided to measure the effect of 6PGD knockdown over glutamine consumption. Indeed, an altered glutamine metabolism was observed when cells were subjected to 6PGD knockdown, since the ratio between glutamine and glucose consumption was higher after 6PGD inhibition (Figure 3C,E), implying that 6PGD knockdown increases MCF7 cells’ glutamine consumption rate. Since we have found 6PGD inhibition to have no noticeable effect on total ROS levels (Figure 3A), we hypothesized that the increased consumption of glutamine could be to produce NADPH via the malic enzyme (ME) and isocitrate dehydrogenase (IDH) to maintain the reductive capacity of the cells which would have been compromised after 6PGD inhibition. Glutamine plays a key role in TCA cycle, such as ME and IDH do, and the fact that 6PGD knockdown increased glutamine consumption together with specific activities of ME and IDH enzymes in MCF7 cells (Figure 3B) is consistent with our hypothesis. It has also been reported that MCF7 cells are more dependent on glutamine than glucose [46], which is also in agreement with our results. (Figure 3E).

### 3.6. 6PGD Knockdown Reduces the Mammosphere Formation Capacity of Breast Cancer Cells

**Figure 4:**
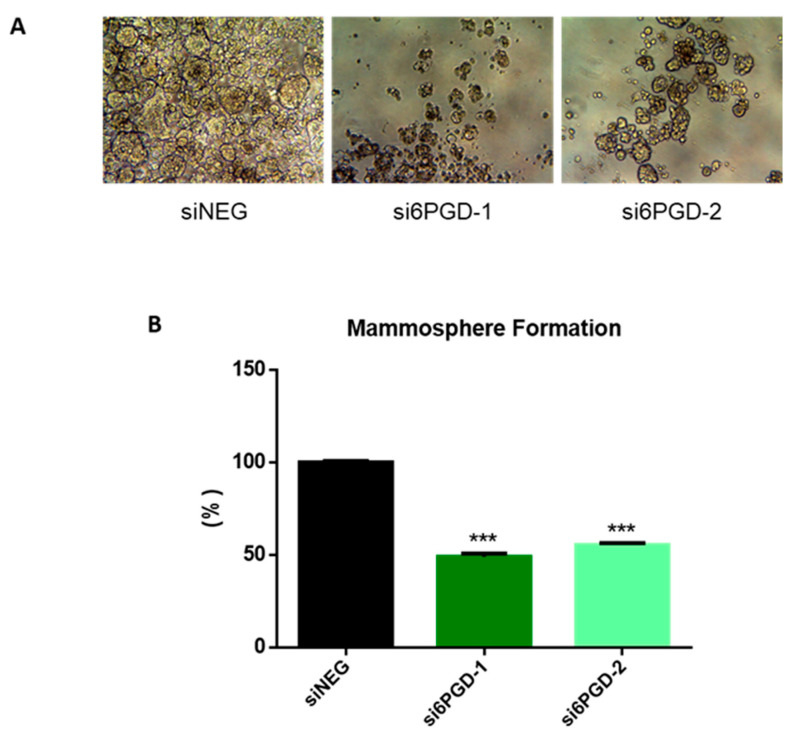
(**A**) Representative photographs showing the different shape, size, and number of mammospheres in each experimental condition. (**B**) Mammosphere formation capability of MCF7 cells after treatment with siNEG or si6PGD. The total mammosphere area for each condition was represented as a percentage relative to the total mammosphere surface area of siNEG-transfected cells. Three asterisks (***) denote *p*-value < 0.001.

One of the biggest challenges in the clinical treatment of tumors is cancer recurrence, a phenomenon occurring due to stem cell-like properties of cancer cells [47]. Therefore, we decided to check whether targeting 6PGD could be an effective method to disrupt the acquisition of stem cell-like properties in MCF7 breast cancer cells. The ability to form single-cell colonies is one of the characteristic features of cancer stem cells (CSCs) [48], so we determined the effect of 6PGD inhibition over the stem cell characteristics of MCF7 cells by measuring their mammosphere formation capability. We observed that mammosphere formation capability of MCF7 decreased around 50% when the cells were transfected with si6PGD compared to the cells transfected with siNEG (Figure 4). Besides, the size of the mammospheres was found to be smaller in cells with reduced 6PGD expression compared to those without 6PGD knockdown (Figure 4A). This indicates that 6PGD has a crucial role in maintaining the stem cell characteristics of these luminal breast cancer cells. The fact that inhibition of 6PGD decreases mammosphere formation capability highlights the potential of 6PGD as a promising therapeutic target.

### 3.7. S3 Is a Specific 6PGD Inhibitor that Induces Senescence in MCF7 Cells

**Figure 5:**
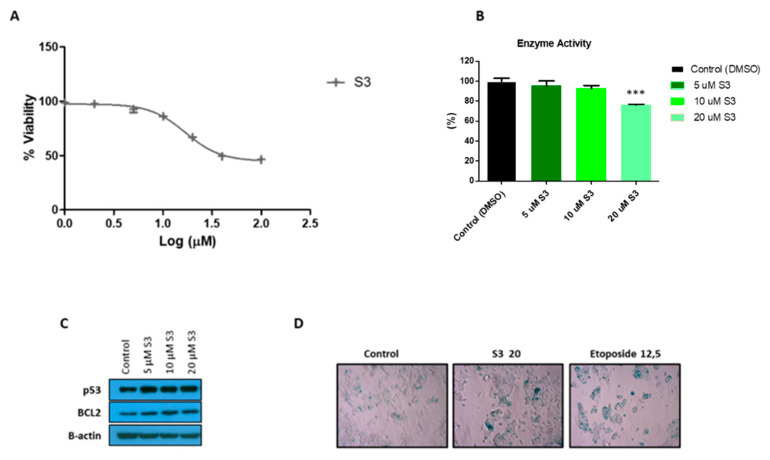
(**A**) Dose response curve of MCF7 cells for selective 6PGD inhibitor S3 after 72 h of treatment of cells with different doses of S3. (**B**) Relative 6PGD specific enzyme activity levels 24 h post treatment with S3. Fold change was quantified relative to control. (**C**) Western blot analysis of p53 and BCL2 expression in cells treated with different doses of S3 or control for 72 h. (**D**) Representative photographs showing increased SA-β-gal positive cells in MCF7 cells treated with 20 µM of S3 compared to cells treated with DMSO alone. In addition, 12.5 µM etoposide was used as a positive control as it is known to induce senescence. Three asterisks (***) denote *p*-value < 0.001.

To further characterize 6PGD inhibition in MCF7 cells, we tested the effects of a selective small-molecule inhibitor of 6PGD, S3 [20]. We first determined the inhibitory effect of this compound on MCF7 cell proliferation (Figure 5A). We observed that around 30 µM of S3 was needed to inhibit the 50% (IC50) of the MCF7 cells. Then, we tested several doses of S3 lower than the IC50 value and checked the impact on the activity of 6PGD in MCF7 cells (Figure 5B). Additionally, 20 µM of S3, which is the IC40 value of S3 in MCF7 cells, inhibited the activity of 6PGD by around 25%. Further, we speculated that inhibition of this enzyme might lead to senescence; thus, we performed an SA-β-gal assay, which is a classic and straightforward method to detect signs of senescence. As shown in Figure 5D, a significant increase in cells positive for SA-β-gal was found in MCF7 cells treated with 20 µM of S3. To confirm these results, we subsequently tested for increased protein levels of the key senescence effectors p53 and the anti-apoptotic protein B-cell lymphoma 2 (BCL-2) (Figure 5C, Supplementary Figure S2B,C). As expected, immunoblot analysis confirmed the activation of the p53 and BCL2 regulatory pathways with even lower doses of S3 treatment. This finding is concordant with the study of Prieur et al. [49].

## 4. Discussion

Our study reveals 6PGD as a key enzyme for MCF7 breast cancer cells with an important metabolic role that is regulated by the p53 tumor suppressor. Several previous studies have highlighted the importance of PPP in breast cancer tumors, especially those in advanced states, such as MCF7 [48,50,51]. Therefore, we focused our attention on investigating the role of the 6PGD, a less studied dehydrogenase enzyme in ox-PPP.

Here, we have first demonstrated that 6PGD has a pivotal role in the proliferation of MCF7 cells. The high dependency of these cells to 6PGD is evident, considering that partial inhibition of this enzyme activity (around 50%) led to about 25% decrease in cell proliferation for MCF7 cells. This finding is in line with a previously reported study carried out by Yang et al. [22]. Several scenarios might be considered in the reduction of breast cancer cells proliferation when 6PGD is impaired. First, decreased nucleotide and NADPH synthesis with inhibition of PPP leads to a decrease in cell proliferation since nucleotides are needed for the biosynthesis of genetic material, and NADPH is required for redox balance and lipid biosynthesis. Besides the increase of glycolysis metabolites, 6PGD inhibition also results in the accumulation of 6-phosphogluconate and 6-phosphogluconolactone, which in turn alters the proliferation of cells [25]. Even though the exact mechanism of action of 6PGD is not totally elucidated, its importance in cell proliferation has been reported in several studies [6,20,26,28,52] and a permanent and stronger knockdown of this gene using shRNA, CRISPR/Cas technology, or small molecules selectively targeting this enzyme is expected to result in even more significant reductions of the proliferation kinetics of the breast cancer cells.

Furthermore, the cell cycle was also altered in 6PGD inhibited breast cancer cells. An expected arrest at the S phase was observed since nucleotide synthesis is partly impaired. Similarly, 6PGD knockdown also induced apoptosis in breast cancer cells. This induction is visible at both the early and late apoptosis/necrosis level. On the other hand, we showed that p53 is augmented on the cells with 6PGD inhibited, which confirms our hypothesis that 6PGD knockdown decreases proliferation of breast cancer cells through p53. Considering that p53 has essential roles in modulation of cell cycle and apoptosis [40] and also knowing that MCF7 cells have wild type p53 [41], the increase in p53 levels resulting from 6PGD inhibition led MCF7 cells to alter cell cycle progression with an arrest in the S phase. Thus, we can conclude that p53 regulates PPP enzymes, including 6PGD. Knowing that AMP-activated protein kinase (AMPK) couples p53 in cell fate decision [53] and that 6PGD ablation alters the AMPK levels in breast cancer cells [22], we can conclude that increase in p53 levels upon inhibition of 6PGD in our experimental design might occur through an AMPK dependent manner.

Next, we observed that 6PGD knockdown did not change the total intracellular ROS level of MCF7 cells. Filosa et al. previously demonstrated that G6PD-knockdown mouse embryonic fibroblasts made use of another source of NADPH production to rescue ROS [54]. We assume that MCF7 with 6PGD inhibition also recover the missing NADPH for redox detoxification from other sources such as glutaminolysis and that the decreased proliferation rate with inhibited 6PGD has an oxidative stress independent mechanism. It has been demonstrated that the G6PD enzyme does not have a strong effect on cellular proliferation but is crucial for defense against oxidative stress [55]; nonetheless, our findings prove the importance of functional 6PGD activity for cell survival in contrast to G6PD activity. On the other hand, Sukhatme et al. showed that 6PGD inhibition increased G6PD activity considerably in lung cancer cells [6]. Even though in our cells, 6PGD knockdown did not cause a strong G6PD activity enhancement, knowing that breast cancer cells have a very active PPP and that especially MCF7 cells have very high G6PD levels, we assume that NADPH produced by G6PD together with enhanced glutaminolysis is enough to compensate the decrease of NADPH resulting from the reduced 6PGD activity as well as to maintain ROS levels.

To have a complete idea about the effect of 6PGD reduction in breast cancer, we assessed some flux measurements related to glucose and glutamine metabolism. Like previously reported studies [6], we also observed a decrease in the glycolytic efficiency after 6PGD inhibition. In addition, the reduction in LDH activity suggests that 6PGD inhibition causes a shift from glycolysis to TCA cycle. On the other hand, 6-PG accumulation has been reported to activate glycolytic enzymes PFK and PK in hepatic cells to carry the excess glucose away from PPP to produce pyruvate for lipid biosynthesis [56,57]. Nevertheless, we observed reduced glucose consumption in MCF7 cells with 6PGD inhibition, which might be due to the fact that glucose metabolism in malignant cells is distinct from that of non-transformed cells.

This study uncovers a link between PPP and glutaminolysis since cells with reduced 6PGD activity consumed more glutamine than control cells. Additionally, the ratio of glutamine consumption rate over glucose consumption rate was significantly increased. As we discussed before, increased glutamine metabolism must be taking place to compensate for both the missing NADPH and nucleotides caused by 6PGD inhibition. Our results also reveal that inhibiting 6PGD entails the upregulation of ME and IDH, which probably constitutes an adaptive mechanism that breast cancer cells undergo to produce NADPH when the ox- PPP has been blocked. Interestingly, this fact reveals an alternative metabolic vulnerability that could be targeted to hamper breast cancer cells’ adaptation to 6PGD inhibition. The simultaneous inhibition of 6PGD and ME or IDH represents a promising strategy to deprive tumor cells of their ability to compensate for the oxidative stress through NADPH production. In fact, a recent publication showed that even though 6PGD enzymatic activity is not indispensable, the inhibition of 6PGD together with any enzyme of non-oxidative PPP (transketolase, transaldolase, ribulose epimerase, or ribulose isomerase) is highly detrimental [58]. That is, targeting other metabolic targets along with 6PGD could result in the development of better therapies against breast cancer cells [59,60].

One of the most striking findings in this study is the uncovered link between PPP and cancer stem cell characteristics. The results provided in this research clearly show that 6PGD inhibition significantly decreased not only the mammosphere formation capability but also the size and number of formed mammospheres. To the best of our knowledge, this is the first study to reveal that an enzyme of the oxidative phase of PPP has significant effect on diminishing the stem cell-like characteristics of breast cancer cells. This could be efficient in preventing breast cancer growth and helpful in addressing the severe problem of cancer recurrence, which is one of the most challenging issues faced by many of the currently targeted therapies. The fact that the inhibition of 6PGD decreases mammosphere formation capability highlights the potential of 6PGD as a promising drug target, and targeting 6PGD could also sensitize cells to current chemotherapeutics, thus improving the efficacy of the most used clinical approaches [61].

Cancer cells are characterized inherently by a dynamic interplay between proliferation, cell death, and/or senescence [62]. Previously, it has been reported that knockdown of 6PGD induces cellular senescence in several cancer types including lung tumors [6]. In accordance with this, our results indicated an increase of SA-β-gal activity in 6PGD-inhibited MCF7 cells. Besides SA-β-gal activity, senescent cells are known to up-regulate p53 [49]. Here, we demonstrated the activation of the p53 following S3 treatment. Similar to apoptosis, senescence also gives opportunities for therapeutic targeting in cancer. The cell fate choice between senescence and apoptosis can be manipulated by targeting upstream mediators of apoptosis, such as the anti-apoptotic Bcl-2 family proteins [63]. Here, we also showed an increased expression of BCL-2 upon S3 treatment in MCF7 breast cancer cells.

Besides our results, a recent study indicated that 6PGD played an important role in the migration of tumor cells in vitro [64]. Results obtained through cell culture might differ from true tumors, as cell culture lines have sustained changes in metabolism that may deviate from the metabolism of native tumors. However, cancer cell lines play a pivotal role in modern cancer research as preclinical model system since they retain most of the genetic properties of the cancer of origin under the right conditions and with appropriate controls. They also have peculiar capability to provide an indefinite source of biological material for gaining mechanistic and therapeutic insight [65]. Therefore, validation of this proof of concept in vivo through xenograft experiments will have to be conducted to further assess the potential of 6PGD inhibition as a therapeutic strategy in breast cancer treatment, as our results highlight the potential of this new approach.

## 5. Conclusions

The experimental evidence presented in this work highlights the potential of 6PGD as a putative therapeutic drug target in breast cancer treatment. Targeting 6PGD not only reduces cell proliferation through cell cycle arrest and apoptosis induction but also activates p53 and decreases the stem cell-like characteristics of breast cancer cells. This could be efficient in preventing breast cancer growth and be helpful in addressing the severe problem of cancer recurrence, which is one of the most challenging issues faced by current targeted therapies. In this sense, targeting 6PGD could also sensitize the cells to current chemotherapeutics, thus improving the efficacy of the most used clinical approaches [61].

## Data Availability

The datasets used and/or analyzed during the current study are available from the corresponding author on reasonable request.

## Supplementary Materials

The following are available online at https://www.mdpi.com/2079-7737/10/2/85/s1, Supplementary Figure S1. Outline of Pentose Phosphate Pathway; Supplementary Figure S2. Intensity ratio of western blots.
