# Supplementary material for: Oxidative Pentose Phosphate Pathway Enzyme 6-Phosphogluconate Dehydrogenase Plays a Key Role in Breast Cancer Metabolism
Source: Biology (Basel). 2021 Jan 23;10(2):85. doi: 10.3390/biology10020085 (PMC7911610; doi:10.3390/biology10020085)
Supplement: Supplementary file 1 [file biology-10-00085-s001.pdf]

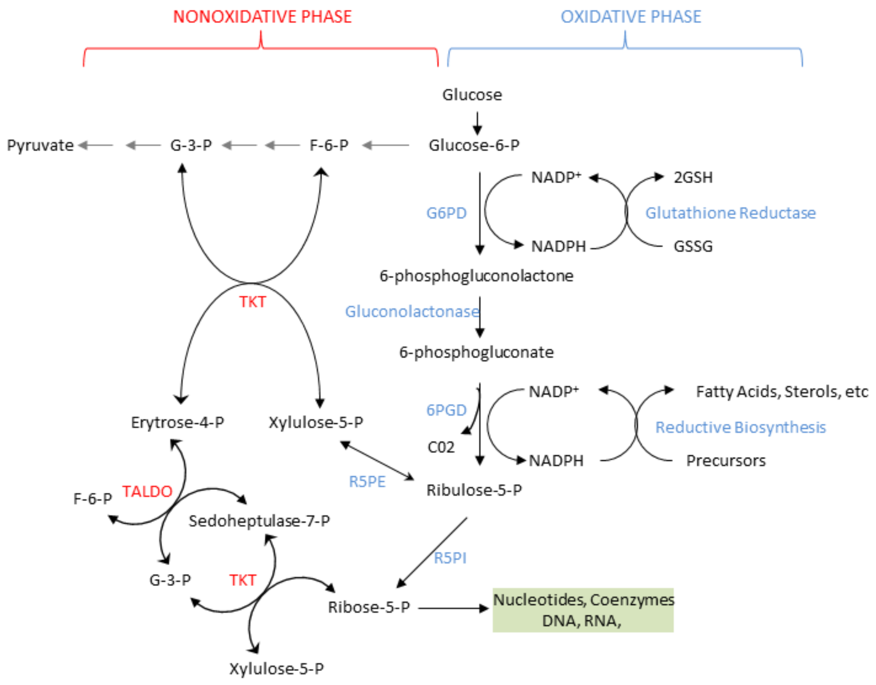

Supplementary Figure S1. Outline of Pentose Phosphate Pathway

The irreversible ox-PPP phase is catalyzed by G6PD, 6PGL (not shown) and 6PGD (6-phosphogluconate dehydrogenase). This phase produces NADPH, used for ROS detoxification, and synthesize fatty acids. The reversible nonoxidative pathway takes role a carbon exchange between PPP and glycolysis, either recycling the excess of pentoses or synthesizing ribose from glycolytic intermediates. The main enzymes involved in this branch are TKT and TALDO. CO2, carbondioxide; E-4-P, erytrose-4-phosphate; F-6-P, fructose-6-phosphate; GSH, glutathione; GSSG, glutathione disulfide, G-3-P, glyceraldehyde-3-phosphate; G-6-P, glucose-6-phosphate; G6PD, glucose-6-phosphate dehydrogenase; NADPH, Nicotinamide adenine dinucleotide phosphate; R-5-P, ribose-5-phosphate; R5PI, ribulose-5-phosphate isomerase; R5PE, ribulose-5-phosphate epimerase; S-7-P, sedoheptulose-7-phosphate; TKT, transketolase; TALDO, transaldolase; X-5-P, xylulose-5-phosphate; 6PGD, 6-phosphogluconate dehydrogenase.

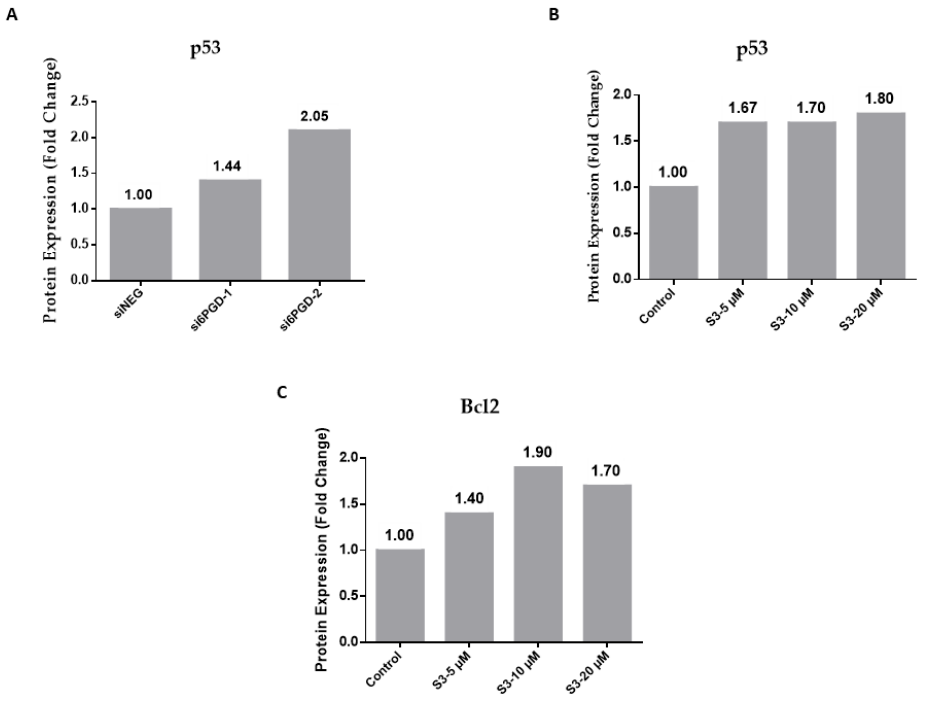

Supplementary Figure S2. Intensity ratio of western blots

Intensity ratio of each band on the western blot was calculated using ImageJ® Software (public domain National Institutes of Health, USA, http://rsbweb.nih.gov/ij/) and normalized to its corresponding actin. **A:** Intensity ratios of p53 in MCF7 cells after treatment of siNEG or siRNA targeting 6PGD. **B:** Intensity ratios of p53 in MCF7 cells after treatment with different doses of S3. **C:** Intensity ratios of Bcl2 in MCF7 cells after treatment with different doses of S3.
